# A novel genetic strategy to enable rapid detection of rare non-native alleles

**DOI:** 10.1038/s41598-024-76149-8

**Published:** 2024-10-29

**Authors:** Robert D. Cooper, Tara K. Luckau, Erin Toffelmier, Dave G. Cook, Stacy Martinelli, Michael H. Fawcett, H. Bradley Shaffer

**Affiliations:** 1 Department of Ecology and Evolutionary Biology, University of California, 610 Charles E. Young Drive East, Los Angeles, CA 90095 USA; 2 La Kretz Center for California Conservation Science, Institute of the Environment and Sustainability, University of California, Los Angeles, CA 90095 USA; 3 3003 Magowan Drive, Santa Rosa, CA 95405 USA; 4 California Department of Fish and Wildlife, Wildlife and Lands Management Program, Santa Rosa, CA 95403 USA; 5 Fawcett Environmental Consulting, 598 South First Street, Dunsmuir, CA 96025 USA

**Keywords:** Genetic hybridization, Conservation biology, Herpetology

## Abstract

Established invasive species represent one of the most harmful and challenging threats to native biodiversity, necessitating methods for Early Detection and Rapid Response. Cryptic invasions are particularly challenging and often require expensive and time-consuming molecular surveys which limits their usefulness for management. We present a novel application of the Fluidigm SNP-Type Assay to identify rare non-native alleles that significantly reduces the cost and time to generate diagnostic results. We demonstrate the efficacy of this method using experimental Fluidigm pools (99% accuracy) and sequence data (96% accuracy). We apply our novel methodology to an endangered population of California tiger salamanders in Sonoma County where two individual non-native tiger salamander hybrids have previously been detected since 2008. We screened 5805 larvae in 387 sample-pools containing 15 larvae each. We did not detect any non-native hybrids in the population, a result that was verified with sequence data, though we strongly recommend additional years of sampling to confirm hybrid absence. Our success with a challenging, large-genome amphibian suggests this method may be applied to any system, and would be particularly useful when it is necessary for conservation practitioners to rapidly identify rare taxa or genes of interest.

## Introduction

The human-mediated spread of non-native taxa harms biodiversity, ecosystem services and human livelihoods, often on a global scale^1^. Increases in trade, travel and transportation homogenize global biodiversity through the establishment of invasive species^2^, resulting in compromised ecosystem stability^3^, decreased public health^4^ and negative impacts on national economies^5^. These impacts highlight the need for rapid and active management strategies that ameliorate the damage caused by non-native species before they become established, given the expense and limited success of large-scale eradication efforts post-establishment^6^. Eradication efforts are further complicated by cryptic invasions, in which non-native species are phenotypically similar to native relatives, and especially when they interbreed with threatened native taxa. When the resulting non-native hybrids are successful and fertile, they can severely complicate management and often lead to an erosion of native genotypes^7–9^. If hybrids do not fill the same ecological role as native taxa, then conservation efforts should focus on containment and removal, provided native genotypes are present or available to recolonize^10^. This strategy is significantly more effective when the invasive species, or their hybrids, are relatively rare and restricted in their distribution. Thus, it is crucial to develop methods for rapid identification and targeted management for incipient invasions, through “Early Detection and Rapid Response” (EDRR) policies^2^.

Cryptic invasions are a pervasive and growing concern in conservation biology. A recent review found that between 1997 and 2016, 70 scientific papers identified cryptic invasions and their threat to native conservation^11^. Many of these systems involve hybridization with native taxa that is recognized using genomic tools, in systems ranging from watermilfoil^12^, marine mussels^13,14^, cyprinid fish^15^, and multiple species of trout^16,17^. Management of these invasions is often limited by the accuracy and/or cost of molecular tools to identify cryptic hybrids. A major challenge across systems is the need to survey multiple, unlinked genetic markers to accurately quantify introgression, especially in backcross generations, as non-native alleles become less common and randomly dispersed across the genome. Here we present a novel, rapid, and inexpensive method to scan large numbers of individuals for rare non-native alleles, as would be the case in the earliest phases of a hybrid invasion. We then apply this method to a population of endangered salamanders threatened by the recent introduction of a non-native congeneric invasive.

The California tiger salamander (“CTS”; *Ambystoma californiense*) is a large-bodied amphibian endemic to central and coastal California. Befitting a member of the mole salamander genus *Ambystoma*, CTS spend the majority of their life in underground rodent burrows, emerging occasionally to feed and breed during winter rain events. Adults breed and lay eggs in ephemeral ponds and the resulting larvae grow and develop over the course of 3–5 months^18^. CTS larvae function as apex predators in occupied vernal pool ecosystems^19^, directly affecting many other endangered vernal pool inhabitants including California red-legged frog (*Rana draytonii*), Santa Cruz long-toed salamander (*Ambystoma macrodactylum croceum*)^20^, several species of fairy shrimp (*Branchinecta spp.*), and vernal pool tadpole shrimp (*Lepidurus packardi*)^19^. CTS larvae then undergo metamorphosis, migrate into upland habitat, sexually mature in about four years, and return to breed once or twice during their 10 + year lifespan^18^.

CTS are listed as threatened throughout their range by the California Department of Fish and Wildlife (CDFW)^21^, while the United Stated Fish and Wildlife Service (USFWS) manages the species as three Distinct Population Segments (DPSs). The most widespread DPS, located in coastal and central California, is listed as threatened under the Endangered Species Act^22^, while the remaining two DPSs, located in Santa Barbara^23^ and Sonoma^24^ Counties, are both listed as endangered. Considerable resources have been allocated to protect these populations from myriad threats including habitat destruction, climate change, and disease. However, the introduction of non-native barred tiger salamanders (“BTS”; *Ambystoma mavortium*) into California has presented one of the most difficult challenges to CTS conservation. Hybrids between CTS and BTS (hereafter “hybrids”) are fertile and appear to enjoy greater fitness than native CTS in both controlled lab environments^25^ and the wild^26–28^, resulting in widespread introgression which threatens to eliminate native genotypes through significant portions of the species’ range. This genetic turnover would have devastating ecological effects on breeding pond biodiversity since hybrid larvae disrupt vernal pool ecosystems, exerting greater top-down predation pressure than native CTS, with cascading effects on everything from amphibian prey to snails and periphyton^20,29^. Currently, there is an extensive CTS/BTS hybrid swarm that is restricted to the Salinas Valley (Monterey County, CA, USA). However, small numbers of hybrids have recently been detected in the Santa Barbara and Sonoma DPSs^30–32^. Particularly in these latter areas where the hybrids are still rare, it is vital to rapidly identify, contain, and hopefully remove these geographically restricted non-native introductions to prevent the further establishment and spread of additional large-scale hybrid swarms.

Rapid identification of hybrids is the critical first step in EDRR strategies. As adults, hybrid salamanders are often visually indistinguishable from native CTS, especially in backcrossed individuals with a low fraction of non-native alleles. Visual identification of hybrids is impossible at all other life stages. These challenges necessitate the use of reliable genomic techniques to identify non-native ancestry. However, the large size of the *Ambystoma* genome (32 Gb)^33,34^ precludes the use of many modern genetic techniques. Past studies have relied on methods including RNA-seq^25^ or using a comprehensive 5,237 gene exon-capture technique^28,35^ to sequence a reduced representation of the genome. While highly accurate, these approaches are expensive and time-consuming, making them impractical for rapid scans of the many individuals required to identify rare hybrids.

**Fig. 1:**
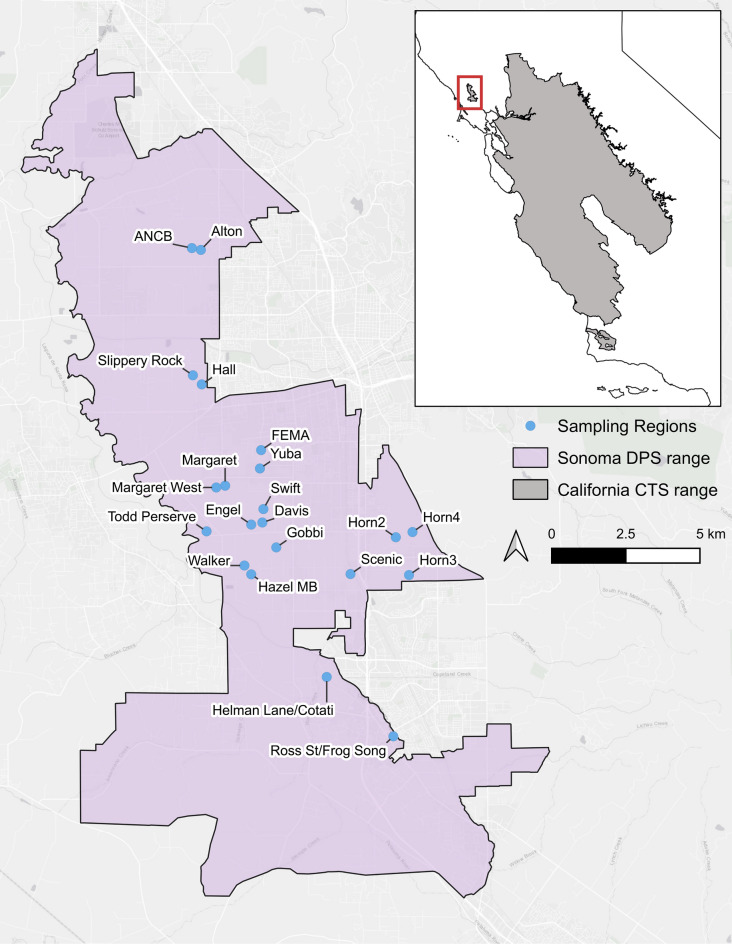
The Sonoma Distinct Population Segment (DPS) of the California tiger salamander (CTS) (red rectangle in range wide inset map) is geographically disjunct and isolated from the central DPS. While every surveyable pond with a known or suspected occurrence was surveyed in the DPS, here we only show regional sampling locations in which CTS were detected in 2019 or 2020. In total, our study includes 72 ponds from 21 regional sampling locations (main map, blue circles) across the Sonoma DPS range.

In this study we develop a novel genotyping method and apply it to an early-stage genetic invasion of the Sonoma DPS. Previous sequencing identified two non-native individuals, one 94% non-native larva and one 48% non-native larva collected from two separate ponds at the Alton Preserve in 2008 and 2014 respectively (Fig. 1). These two samples represent only 0.4% of the 479 samples that have been analyzed from the Sonoma DPS from 1989 to 2018 (Shaffer et al. unpublished data), and suggest that hybrids with non-native BTS, if still extant, are rare at this early stage in the DPS. Given this predicted low prevalence, we developed our novel technique and applied it to facilitate EDRR removal programs to prevent an even more devastating hybrid swarm from establishing in the endangered Sonoma population.

We present a novel application of the Fluidigm SNP-Type Assay (now Standard Bio Tools: store.standardbio.com), optimize it for a set of diagnostic loci for rapid detection of non-native alleles, and apply it across the Sonoma DPS to a comprehensive collection made in the 2019 and 2020 breeding seasons. The assay technology uses allele-specific extension and endpoint fluorescent detection on the Fluidigm nanofluidic and imaging platform, enabling a low-input, high-throughput, and cost-effective workflow. Our method inputs pools of individuals for detection of non-native alleles within the sample-pool, which is a novel application of the Fluidigm technology. This study has two main objectives: 1) describe a novel method for the rapid detection of specific alleles in large sample sets and 2) apply this method to identify the extent of non-native alleles in the endangered Sonoma CTS population. We describe the process for development and validation of the detection assay in considerable detail so that it may be applied to other taxa and a range of conservation and management applications.

## Methods

### Field sampling

Field collections were conducted in the spring of 2019 and 2020. We attempted to sample most sites with suitable CTS breeding habitat and every surveyable pond that had previously held CTS within the Sonoma DPS. We generated a map (Fig. 1) of the DPS and all sampled sites using QGIS (v3.30.1; QGIS.org)^36^. Polygons for each distinct population segment boundary were developed by the California Department of Fish and Wildlife and hosted by the California Open Data Portal^37–39^. Ponds are typically < 0.5 m deep and easily accessible to wading surveyors. We collected larvae using D‐shaped dipnets, with 3.2 mm mesh, swept along the pond bottom. In larger ponds, collections were supplemented with a 5 m-long, 3.2 mm mesh seine that was drawn through the water column. CTS were held in buckets filled with pond water, sampled by harvesting the distal 5–10 mm of each larval tail in the field using scissors, and released immediately after tissue collection. Individuals were held for no more than one hour, and tissue samples from each pond were stored together in one or more 15 ml or 50 ml conical tubes filled with 95% ethanol. These field collection methods and all other experimental methods reported here follow the “Animal Research: Reporting of in Vivo Experiments” (ARRIVE) guidelines^40^. All methods in this study have been reviewed and approved by both the USFWS and UCLA Animal Research Committee (ARC-2013–011). All experiments were performed in accordance with relevant guidelines and regulations.

### DNA isolation

To rapidly screen thousands of individuals, we used a pooled sample approach. To facilitate consistent tissue input, and therefore consistent allelic representation for each individual in the multi-sample-pool (hereafter, “DNA-pool”), we used a 3 mm diameter biopsy tool (Electron Microscopy Sciences) to excise a circular tissue punch from the proximal section of the harvested tail tissue including as much of the caudal musculature as possible. We combined up to 15 punches from larvae that were collected from the same pond in a single DNA-pool. If there were fewer than 10 tissues available for a given DNA-pool, we combined tissues from two ponds to create mixed-pond DNA-pools. DNA-pools were extracted on a Kingfisher 96 Extractor (ThermoFisher) using MagMax DNA Isolation Kits (ThermoFisher) following the manufacturer’s protocol.

### Nanofluidic SNP type assay

We used the 96 × 96 Fluidigm (now Standard Bio Tools) SNP-type assay, which employs an integrated fluidic circuit (IFC or “chip”). This chip uses nanofluidics to assay 96 samples for 96 SNPs, yielding 9216 individual 6.75 nL reactions. In our application, each sealed reaction consists of a single SNP assay and a single DNA-pool which are activated through thermal cycling. However, rather than assay 96 unique SNPs, we scored 48 SNPs in duplicate across the 96 SNP panel to serve as technical replicates. Upon completion of the assay, an endpoint fluorescent image is captured on the Fluidigm EP1 system, which compares excitation and emission peaks for each allele-specific reporter (FAM and HEX) against the background ROX emission. Using the Fluidigm SNP Genotyping Software, previous studies genotyping individual organisms reported a 99–98% accuracy in calls that were verified using alternative sequencing techniques^41^.

We developed CTS/BTS diagnostic Fluidigm assays for the Sonoma DPS using an established 5237 target exon panel^35^. We followed previous protocols^28^ to identify diagnostic SNPs differentiating CTS collected from across the Sonoma DPS and BTS from the same population that was introduced into California^42^. From this set we selected regions with a single diagnostic SNP flanked by at least 60 invariant bases on either side with GC content under 65%, resulting in 111 potential target regions. We sent these potential targets to the D3 Array Design team at Fluidigm (now Standard BioTools), who created 56 potential Fluidigm Assays. We then ran diagnostic tests and selected the 48 best performing Assays based on fluorescent intensity and genotype accuracy across a set of known CTS and BTS controls using the standard Fluidigm Genotyping Software. A full description of these assays are provided in the supplemental materials (SI.1).

### Experimental optimization

Given that the Fluidigm assay is designed for individual genotyping rather than detecting a rare allele in a pool of individuals, we performed several experiments to optimize our detection of BTS alleles while minimizing the number of assays to reduce cost and time. We determined the optimal amount of total input DNA by running Fluidigm assays on a range of input DNA quantities and compared the relative fluorescence of known heterozygous samples (see SI.2). We conducted experiments to determine the greatest number of individuals that could be pooled, while still providing sufficient sensitivity to detect a single hybrid in a pool of otherwise all-CTS genotypes (see SI.3). We define the fraction of BTS allelic copies in a DNA-pool as the Rare Allele Ratio (“RARatio”). For example at a given locus, one heterozygous larva (1 BTS and 1 CTS allele) combined with 14 homozygous CTS larvae (2 CTS alleles each, 28 CTS alleles total) would amount to a RARatio of 1/30, or $0.0\overline{3 }$. Our goal was to identify the RARatio that is as small as possible while still being able to unambiguously differentiate it from a DNA-pool with no BTS alleles (RARatio = 0).

### Random forest custom genotype calls

Given our pooled DNA sampling design and non-standard use of the Fluidigm platform, we developed a custom statistical strategy to identify non-native alleles in sample DNA pools. We used known-genotype DNA-pools at specific RARatios to train a Random Forest (RF) Classification Model using the software package randomForest (v4.6-14)^43^ in the statistical language R (v4.0.4)^44^, using the observed intensity of X-Allele and Y-Allele fluorescence for each SNP-assay to estimate the presence or absence of BTS alleles at that locus. The model was trained using multiple independent chips with sample pools comprised of native CTS DNA from Sonoma, combined with a small portion of BTS DNA in 1:15 and 1:30 RARatios. Importantly, these DNA-pools were constructed by adding precise amounts of pure CTS and BTS DNA together, not by using tissue punches from known-genotype individuals. Thus, in these initial experiments, the added potential variation from actual tissue sampling was not included. We randomly assigned half of these known-genotype pools to train the model, reserving half to evaluate model accuracy.

We modeled each SNP using two separate sets of predictor variables: Raw X- and Y-Allele intensity and control-corrected X- and Y-Allele intensity. The raw allele intensity was the direct output of the Fluidigm software, where allelic intensities were automatically corrected using the negative controls. The control-corrected allele intensities were manually calculated by subtracting the chip-specific background allele intensity, estimated as the mean intensity of the absent allele that was scored as present (generally, a very low value derived from background reflectance) in each set of controls. That is, for each chip we took the average value of the BTS allele (Y-Allele) in the 100% CTS control and subtracted that from all the samples on that chip, assuming that this level of ‘false positive” fluorescence was uniformly present for every SNP. Similarly, we took the mean value of the CTS allele (X-Allele) in the BTS control and subtracted that from all samples on that chip. This approach allowed us to partially control for baseline differences in allele intensity between specific assays and IFC chip runs. We used these baseline intensities to quantify and compare the amount of variation between IFC chip runs and between different SNP-assays using ANOVA tests.

We evaluated the RF models by comparing estimates of the ‘Out of Box’ error (OOB), call probability, sensitivity, specificity, and overall model accuracy. The OOB error represents the uncertainty level in the RF model, which is estimated by excluding a small subset of the data and evaluating the degree of change in model prediction for each RF iteration. We report the median and interquartile range of OOB error across all SNP-specific RF models. Call probability represents the RF model confidence for each genotype call, which ranges from 0.5 to 1, where 1 is 100% confidence and 0.5 or 50% is complete lack of confidence. These probabilities were generated for each genotype using the predict (…, type = “prob”) function in R. Sensitivity quantifies the ability of the model to detect true BTS alleles in hybrid sample-pools (“True Positive Rate”). This was calculated as the number of pools with BTS alleles identified by the model, divided by the true number of pools with BTS alleles. Specificity represents the ability of the model to correctly identify the absence of BTS alleles in all-native CTS DNA- pools (“True negative rate”) and was calculated as the number of DNA-pools identified as all-native (no BTS alleles detected), divided by the true number of all-native DNA-pools. Accuracy is defined as the proportion of all DNA-pool genotype calls that are correct (i.e. correctly identifying both pools with rare BTS alleles, and pools with all CTS alleles). To estimate sensitivity, specificity, and accuracy we applied the SNP-specific RF model to the remaining half of the known-genotype DNA-pools not included in the model training. We also used these metrics to compare model performance for the raw and control-corrected allele intensity using paired t-tests. We apply Bayesian proportion tests from the R package BayesianFirstAid^45^ to each SNP-specific RF model to explicitly estimate the probability that each genotype call is correct based on the number of false-positives and false-negatives in our training data.

Following these tests, we eliminated SNP assays that failed to accurately detect non-native alleles in known-genotype DNA-pools. We dropped all SNPs with an OOB error rate greater than 5% or a sensitivity less than 90%. Conservatively filtering these unreliable SNPs improved the overall accuracy of our genotype calling, which should result in fewer erroneous calls in the field-collected samples.

### Evaluating accuracy with exon-capture data

To quantify the accuracy of Fluidigm genotype calls, we applied this method to DNA-pools containing DNA from Sonoma CTS mixed with small amounts of DNA from known hybrid animals from Monterey County, CA. We then generated genomic sequence data for all included hybrids using an established exon-capture protocol^28,35^. These hybrids may contain any combination of alleles at a particular target locus (homozygous BTS, homozygous CTS, or heterozygous BTS/CTS), accurately representing the expected diversity in wild hybrid populations. We evaluated the success of the Fluidigm assays at detecting BTS alleles in multiple types of experimental DNA-pools that differ in their method of pooling, RARatio, and final DNA concentration. The first pooling method consisted of combining known concentrations of already-extracted DNA to create specific RARatio pools (“extract-pools”), and the second method consisted of combining specific ratios of tissue, before DNA extraction, such that the pool of combined tissues was extracted together (“tissue-pools”); this latter approach includes variance due to tissue sample size and DNA content, and accurately represents the pooling strategy applied to all field-collected samples. The final factor concerned the degree of dilution of the DNA post amplification in the Fluidigm protocol. The protocol involves diluting the amplified DNA by 1:100. We wanted to test whether over-dilution may have affected call accuracy, so we added a treatment that diluted 1:200. Therefore, the 6 treatment levels included: 1) Extract-pool RARatio 1:1, essentially unpooled DNA from a single hybrid; 2) Extract-pool RARatio = 1:8 (1 part hybrid DNA: 7 parts Sonoma CTS DNA); 3) Extract-pool RARatio = 1:15; 4) Extract-pool RARatio = 1:20; 5) Tissue-pool 1:15 (1 hybrid tissue punch combined with 14 CTS tissue punches all extracted together, RARatio = 1:15–1:30); and 6) Tissue-pool 1:15 that was diluted 2x (1:200 instead of 1:100).

We then evaluated the accuracy of the RF genotyping model by constructing a generalized binomial mixed-effect model (GLMM) using the R package “lme4”^46^. We defined the binary response variable as 1 if the presence/absence of a BTS allele was correctly assigned, and 0 if it was incorrect. We included RARatio, dilution (1 × or 2x) and locus zygosity (number of BTS allele copies at the specific locus) as scaled continuous predictors and pool type (extract or tissue) as a discrete predictor, with hybrid ID and SNP-assay as random effects. We report overall accuracy of RF genotyping along with the effect of each treatment type.

### Genotyping field-collected samples

We assayed up to 75 wild-caught Sonoma CTS larvae from each pond in DNA-pools of up to 15 individuals to screen for the presence of BTS alleles. Each Fluidigm IFC run included 6 controls: 2 known BTS control samples (“BTS controls”), 2 known CTS control samples (“CTS controls”) and 2 negative controls (“NTC”) containing no DNA. For each DNA-pool, we included a technical replicate in the run such that each of 48 SNPs was assayed twice on the same chip for each DNA-pool. We then found the absolute difference in X- and Y-Allele intensity between the two technical replicates. We analyzed this distribution for each allele and removed all assay pairs that exhibited a difference of greater than one standard deviation. This removed spurious results that were likely artifactual, reducing the number of erroneous genotype calls. Next, we used our SNP-specific RF models to call DNA-pools as either heterozygous (“Het”, containing any BTS allele) or homozygous CTS (“CTS”, not containing any BTS alleles) at each SNP locus.

Given unavoidable small error-rates in the RF model and genotype assignment, and the extent to which we may be pushing the technical envelope of the platform, we expect some false positive Het calls. Since a true Het genotype should consistently be identified as a Het across both technical replicates, we filtered out those where only one of the two SNP-assay technical replicates was called as Het, and considered the remaining pools to be “suspect pools” for further validation using three independent methods. While we do not believe that all of these validation steps are necessary for future applications of the method, we include them here as additional tests of our approach and to detail alternative methods for validation that future studies may wish to adopt.

First, we re-assayed all suspect pools using the same Fluidigm-RF genotyping pipeline to determine if the first Het call was repeatable. Second, we evaluated all suspect pools by sequencing the 48 Fluidigm assay regions to validate the presence of non-native alleles. We re-amplified the Fluidigm regions by performing a second Specific Target Amplification (STA), which is used in the standard Fluidigm protocol to amplify the general SNP-assay regions from genomic DNA using multiplex PCR. These amplicons were then cleaned and prepared into Illumina compatible DNA libraries using established protocols^28,35^. Sequence data were mapped to the 48 SNP targets, and the number of CTS and BTS allele counts were reported at each locus for all samples. We included 31 additional DNA-pools as redundant verification to confirm that no hybrid alleles were detected in the same or nearby ponds where the two hybrids had been detected in the past. We also included a hybrid individual from Monterey County (Sample “1600”, 80.6% BTS based on earlier sequencing) sequenced in four DNA-pool sizes as a positive control. Third, DNA-pools were also analyzed by re-extracting DNA from each individual sample that comprised the pool and running them through the standard Fluidigm protocol. Any individual samples that were confirmed as hybrid in any of these three validation analyses are considered true hybrid samples. If no validation method identified non-native alleles, we consider the initial Fluidigm Het call for the suspect pool to be an erroneous false positive.

## Results

### Field sampling

Exhaustive field surveys of 334 ponds in 2019 yielded 13,085 tissues from 108 ponds distributed across 21 out of 36 sites in the Sonoma DPS (Fig. 1). Below average rainfall in 2020 yielded significantly fewer samples, with only 94 tissues collected from 4 ponds at a single site, Alton, where the two non-native hybrid individuals were collected in 2008 and 2014.

### Random forest custom SNP calls

**Fig. 2:**
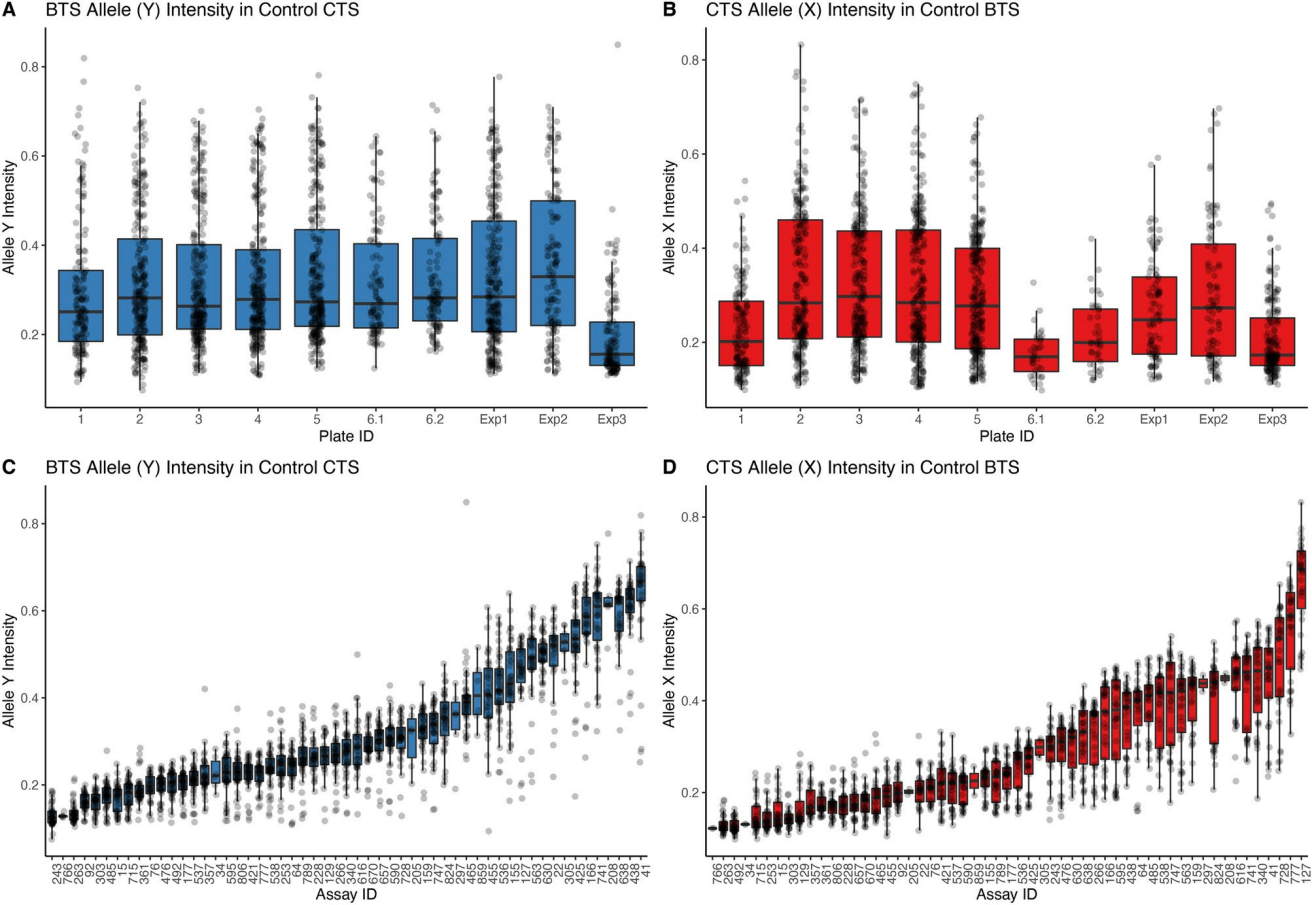
Differences in the fluorescent intensity of Allele X and Allele Y across independent Fluidigm IFC runs (“Plates”; **A** and **B**) and across SNP-Assays (**C** and **D**). Panels A and C depict the intensity of the BTS allele (Y) in control CTS (X) homozygotes (XX), while panels B and D show the CTS allele (X) intensity in control BTS (Y) homozygotes (YY). These values were used to quantify the “background” intensity in samples where we should not see any fluorescence, since each control individual is homozygous for the absent allele. There is minor, but statistically significant variation in background intensity across plates. There are greater and consistent differences in background fluorescence across SNP-Assays.

We explored the amount of background allele intensity in control individuals that do not contain the allele of interest (i.e., intensity of allele X in a homozygous YY individual). We found significant differences in background intensity between IFC chips for the BTS alleles (ANOVA: *F* = 19.69, df = 12, effect size ${\eta }^{2}$= 0.09, p < 2e^−16^; Fig. 2A) and the CTS alleles (ANOVA: *F* = 15.11, df = 12, ${\eta }^{2}$= 0.06, p < 2e^−16^; Fig. 2B). We also detected significant differences in intensity between SNP-specific assays for the BTS allele (ANOVA: *F* = 207.7, df = 54, ${\eta }^{2}$= 0.82, p < 2e^−16^; Fig. 2C) and the CTS allele (ANOVA: *F* = 245.3, df = 54, ${\eta }^{2}$ = 0.82, p < 2e^−16^; Fig. 2D). Differences between SNPs had a much greater effect on allele intensity than chip differences for both the BTS (0.82/0.09 = 9.1 × greater ${\eta }^{2}$) and the CTS (0.82/0.06 = 13.7 × greater ${\eta }^{2}$) alleles.

**Fig. 3:**
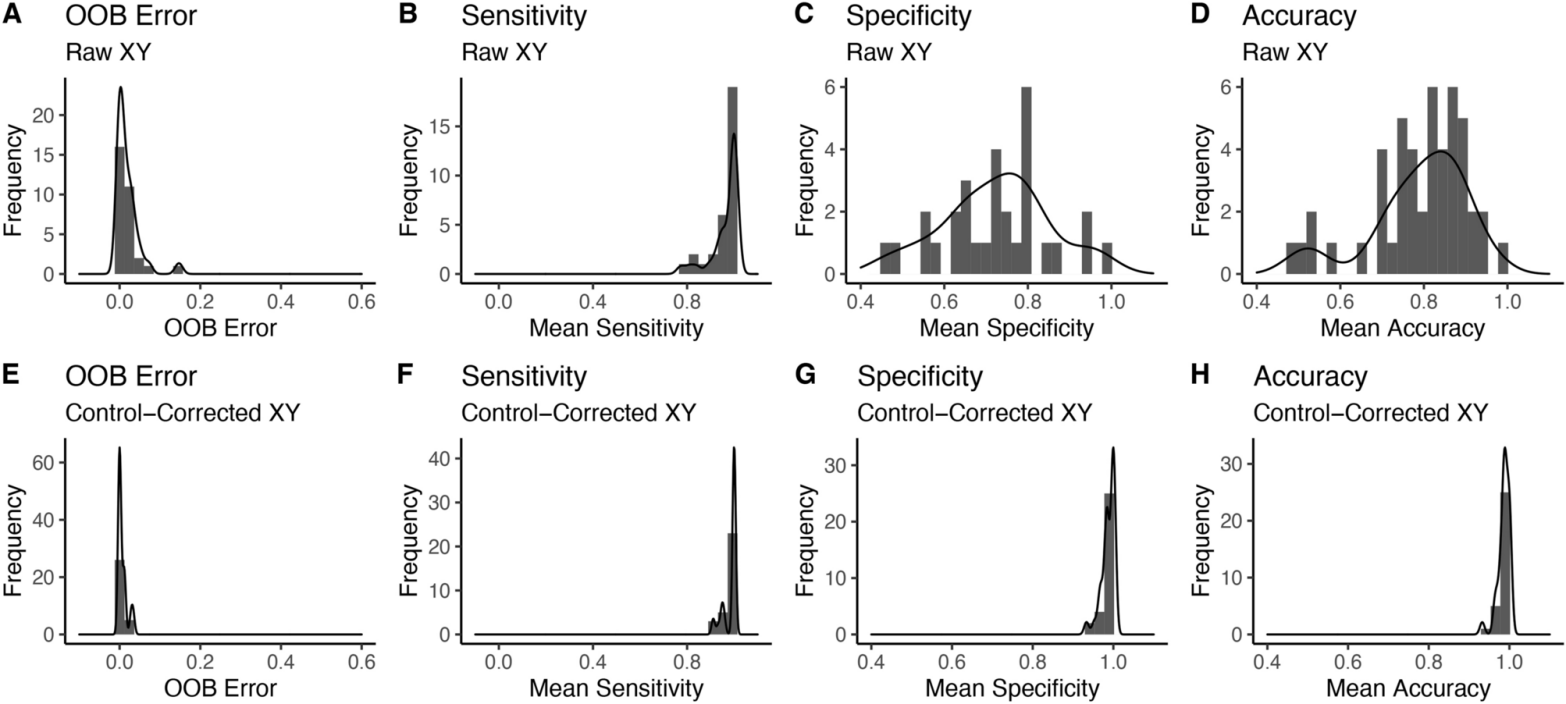
Histograms showing raw (**A**−**D**) and control-corrected (**E**−**H**) allelic intensity variables, and their effect on model out-of-bag error, sensitivity, specificity and overall accuracy. Each SNP-specific Random Forest model was trained using a subset of the data (50%), then tested on the remaining experimental data (50%) distributed across multiple IFC chips. Sensitivity estimates the true-positivity rate (correctly identifying Hets), specificity estimates the true-negativity rate (correctly identifying homozygous CTS) and accuracy is an overall measure of correct genotype call. The control-corrected intensities yielded 21% greater overall accuracy (Paired t-test: t = −7.95, df = 47, p = 2.98e−10; panel H versus panel D).

**Fig. 4:**
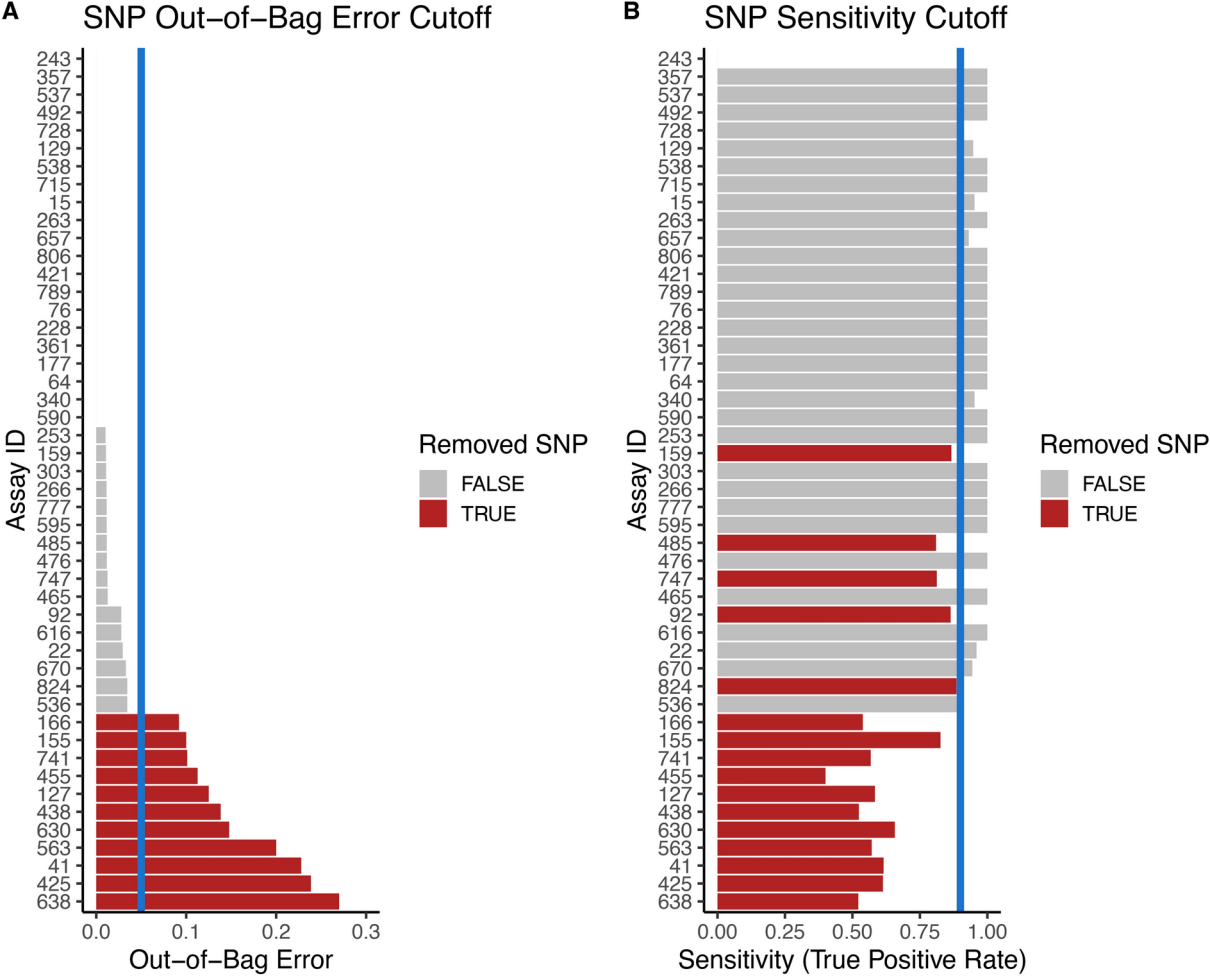
Horizontal bar plots depicting the out-of-bag (OOB) error and sensitivity for each SNP. Blue vertical lines signify filtering thresholds. SNPs were removed from the study if their OOB error was greater than 0.05 (5%) and if their sensitivity was less than 0.9 (90%). Red horizontal bars highlight the SNPs that failed filtering and were therefore not used to call genotypes. A total of 31 SNP assays passed both filters and were used in the final analyses.

We compared the fit of the Random Forest model using Raw X- and Y-Allele intensity and control-corrected X- and Y-Allele intensity to select the most robust method for genotyping unknown DNA-pools. Using the control-corrected intensities improved the accuracy by 21% (Paired t-test: *t* = −7.95, df = 47, p = 2.98e^−10^; Fig. 3D,H). SNP-assays were filtered using cut-offs for OOB error and sensitivity. We identified and removed 11/48 SNPs that had an OOB error greater than 5% (Fig. 4A) and 17/48 SNPs that had a sensitivity of less than 90% (Fig. 4B). These were fully overlapping sets, and in total, we dropped 17/48 SNPs from the analysis, ensuring greater confidence in downstream genotype calls. After filtering the RF model had an OOB error of 0% (95% Confidence Interval = 0–1.1), a sensitivity of 100% (98–100), a specificity of 98.5% (98.3–100), and an overall accuracy of 98.8% (98.3–100). This translates to a 0% false negative rate and 1.2% false positive rate. Applying Bayesian proportion tests across these SNPs yielded the median probability of a locus having BTS alleles given a positive test of 96.9% (95% Credible Interval = 87.5–100), and the probability of a negative test truly lacking BTS alleles of 97.5%; (CI = 93–99).

### Evaluating with exon-capture data

Comparing the genotype calls produced using our custom RF model with sequence data demonstrated the efficacy of our new methodological approach. For samples in a DNA-pool size of 15 the model had a sensitivity of 96.1%, a specificity of 66.7%, and an overall accuracy of 95.6%. The Bayesian proportion test yielded similar estimates: a DNA-pool that is genotyped as all-CTS is correct 95% (95% Credible Interval = 94–96) of the time, whereas a DNA-pool that is identified as het has a lower success ratio at 65% (53–76). The lower specificity in this validation presumably results from tuning the model to maximize sensitivity, since it is preferrable to flag all potential BTS-allele-containing-pools and further validate that call rather than missing true het pools in the initial screening. This result may also stem from the relatively few test genotypes that were homozygous CTS (24 XX vs 1494 XY), this lower sample size may result in greater stochastic variability. Regardless, the overall accuracy was very high, with a Bayesian probability of correctly genotyping a sample at 96% (95–97). RARatio was positively correlated with call accuracy ($\beta$ = 1.48, df = 3273, *z* = 4.35, p = 1.39e^−5^), confirming that smaller RARatios (derived from larger pool sizes) decreased accuracy. Locus zygosity ($\beta$ = 0.67, df = 3273, *z* = 6.83, p = 8.26e^−12^) was positively correlated with call accuracy, meaning that two copies of the BTS allele (i.e. homozygous BTS) improved call accuracy over a single copy (heterozygous for BTS allele). The method of DNA-pooling also affected call accuracy ($\beta$ = 2.16, df = 3273, *z* = 4.60, p = 4.26e^−6^) suggesting the somewhat surprising result that samples pooled using tissue plugs performed better than samples pooled as extracted DNA. Dilution of the post-amplification product did not appear to affect call accuracy ($\beta$ = 0.08, df = 3273, *z* = 0.412, p = 0.68).

### Genotyping field samples

Out of the 13,085 larval samples collected across 2019–2020 we genotyped a total of 5,805 individuals combined into 387 sample-pools. This resulted in 24,700 individual genotype calls after quality filtering, which is comprised of 13,219 unique SNP × DNA-pool combinations. Of these 24,700 genotype assignments, the RF model identified 37 potential Heterozygous (Het) SNP x sample assays, representing less than 0.1% of calls. Het calls were distributed unevenly across 12 SNPs (Chi-squared: X^2^ = 51.5, df = 11, p = 3.3e^−7^). Amongst SNPs that contained potential Hets, there was a significant, negative relationship between number of het calls and SNP specificity (Linear Regression: $\beta$ =  − 4.12e^−3^, *t* =  − 3.37, df = 10, p = 0.007).

**Fig. 5:**
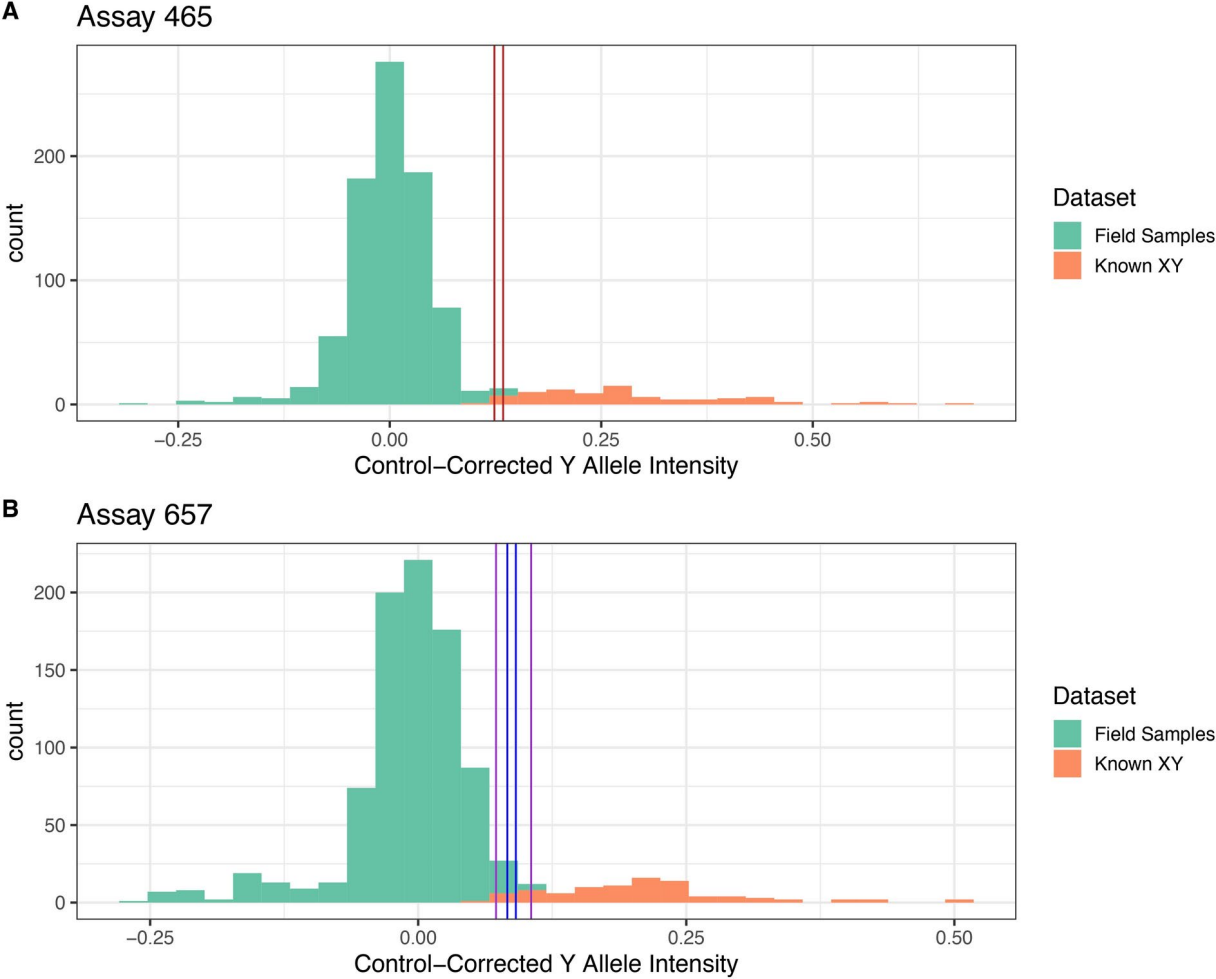
BTS allele intensity of the three DNA-pools with potential non-native alleles detected. Histograms show the distribution of control-corrected Y allele (BTS allele) intensities for the two SNP assays where potential BTS alleles were detected. Two datasets are shown here: DNA-pools consisting of field-collected samples which are of unknown genotype but presumed to be homozygous X (CTS allele) and the artificially constructed heterozygous DNA-pools which are known to be heterozygous XY (CTS:BTS alleles). The vertical lines depict the Y intensity values of the three suspect pools, each color represents a different sample (Red = “E-Hn2-002”, Purple = “Slp-8-C”, Blue = “D-Hn4-008”), and each sample has two lines showing the two assay replicates for each run. The location of all of the suspect pool Y intensities, which overlap both the distribution of XX and XY genotypes, support the conclusion that these calls are spurious, resulting from high fluorescence, but within the reasonable distribution of XX pools.

**Fig. 6:**
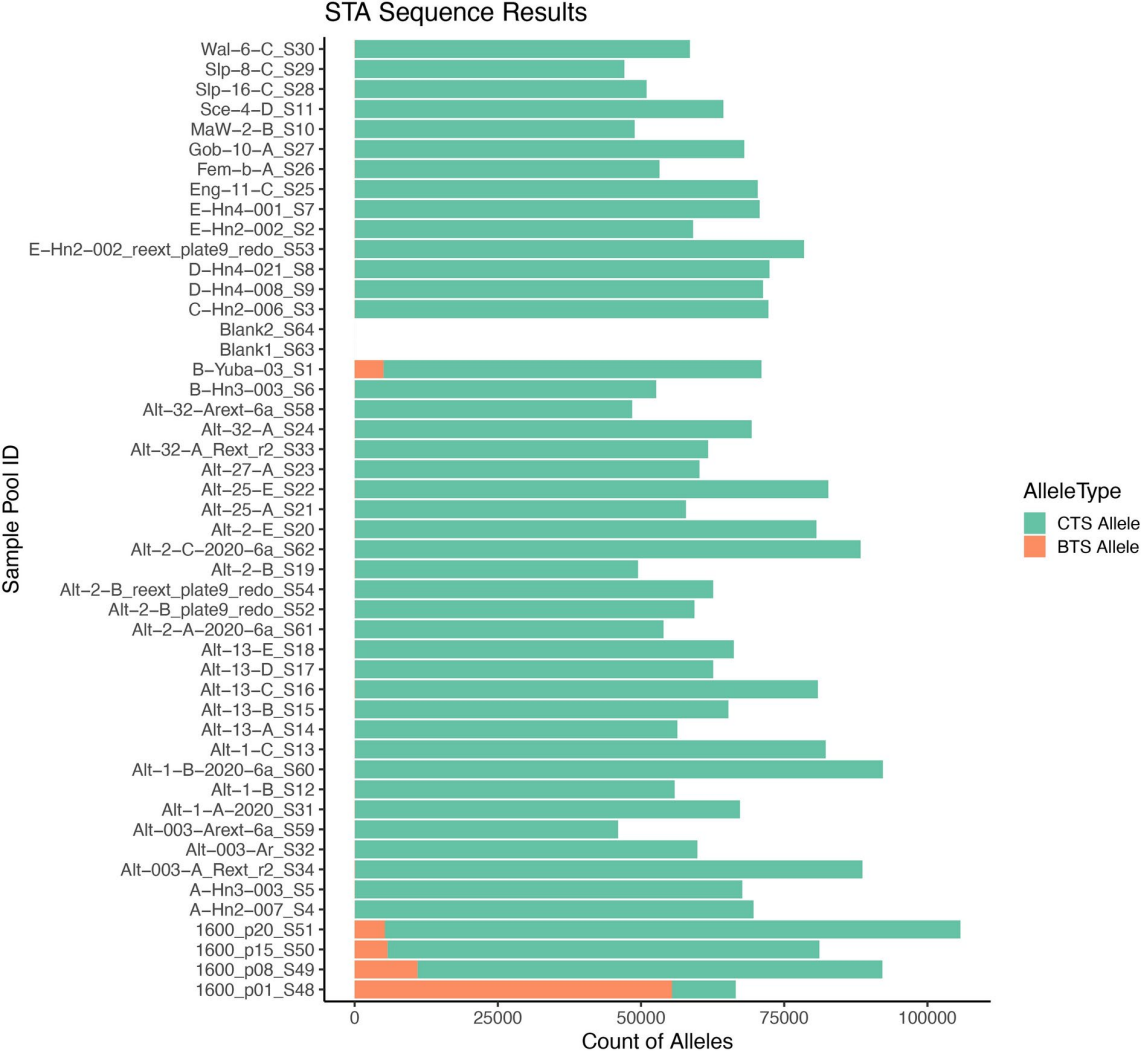
Horizontally stacked bar plot showing the counts of CTS (green) and BTS (orange) alleles in the STA amplicon sequence results. Samples beginning with “1600” had wild-hybrid DNA from Monterey County intentionally introduced into the pool. Individual 1600 was included in the “Evaluating with exon-capture data” section of the manuscript and is 80.6% BTS. This individual was sequenced in four separate DNA-pools that span a range of RARatios designated by “p20”, “p15”, “p08”, and “p01”, which correspond to this single hybrid in a pool of 20, 15, 8, and 1 individuals, respectively. Blank1 and Blank2 were negative control samples. Sample-pool “B-Yuba-03” appears to have BTS alleles, though this is likely due to sample contamination followed by PCR, as no other analysis detected BTS alleles in this sample, or this pond in general (See Discusion).

These 37 Het calls map to 33 unique sample pools distributed across 24 ponds and 11 sites. We removed 34/37 (91%) sample pools where the RF model only identified one of the two assay replicates as heterozygous, and retained three sample pools that were most likely to contain non-native alleles (Fig. 5). Each of these sample pools was assayed again on a separate IFC chip, and no BTS alleles were detected in this replicate run (See SI.4). Although we were unable to reproduce any of the Het calls from the suspect pools, we proceeded to interrogate these pools using two additional and independent validation methods. First, we performed amplicon sequencing on the STA products and counted the number of CTS and BTS alleles observed. We achieved an average sequence depth of 579 reads per SNP target region, which provides sufficient depth to detect BTS alleles at very low RARatio; if a single heterozygous individual was present (RARatio of 1:30) we would expect to sequence 19 copies of the BTS allele (Fig. 6). We sequenced four positive-control pools with a known hybrid included in various pool sizes to confirm the sensitivity of this approach. BTS alleles were detected in each of these positive-control pools at ratios consistent with the pool size (Fig. 6). We did not detect BTS alleles in any of the three suspect pools, confirming the absence of non-native alleles. Across the 42 wild-larval sample pools that we sequenced, we detected BTS alleles in only one pool, “B-Yuba-03”, which is likely a technical artifact. This sample was included as a random addition to the STA amplification to confirm negative results, as no other methods identified BTS alleles in this DNA-pool, or in the five other DNA-pools created from this pond. While we take extensive precautions against sample contamination, we assume that this DNA-pool became contaminated with small amounts of control BTS DNA. The sequence data confirm this hypothesis, since BTS alleles were detected at every locus, a virtually impossible situation in this wild population, and one that would have been detected on the Fluidigm. We are therefore confident that this is not a true BTS detection. Second, we individually extracted tail clips from each of the three suspect pools to demonstrate an alternative validation method using the standard Fluidigm genotyping software. We did not detect any BTS alleles in any of the 45 individuals assayed, however 3 samples had low fluorescence due to insufficient DNA after extraction, stemming from insufficient tissue harvested in the field. This low input DNA may have produced the spurious Het call in the original sample pool as well.

## Discussion

Our primary goal in this study is to introduce a novel use of the Fluidigm SNP-Type assay to scan for rare alleles in any system where rapid genotyping is necessary. By extensively testing it with an actual field study of the endangered Sonoma CTS, we also use it to advance EDRR in the tiger salamander system. The ability of our new pipeline to screen orders-of-magnitude more individuals than a standard sequencing approach enables hybrid detection even at trace levels. Our method, once optimized, takes 3 days to complete whereas traditional exon-capture and bioinformatics methods take approximately 90 days, reducing time-to-results 30-fold. For EDRR, timing is critical, especially when organisms are only present and detectable for a limited time on the landscape. For CTS, this rapid screening enables potential management strategies that target hybrid removal at the pond or individual level in less than a week. We do not believe that this method should replace traditional sequence approaches, and in fact those latter approaches remain the gold standard for confirmatory genotyping. Rather, we advocate it as an additional tool to advance EDRR and facilitate the management of non-native organisms.

Using known, homozygous samples (100% CTS and 100% BTS) we were able to quantify the background allelic intensity in samples that did not contain the focal allele. There were large, consistent differences in the background fluorescence between each SNP assay. Although we are not sure why they exist, these differences may arise from the multiplexed PCR step in the Fluidigm Protocol for non-model organisms, which is used to increase the target sequences relative to the non-target input DNA. Some target primer pairs may preferentially amplify, achieving a greater relative frequency prior to the SNP-specific amplification and fluorescence^47^. To counter this artifact, we constructed SNP-specific statistical models to call genotypes. We also detected slight differences in background fluorescence across Fluidigm chip runs. These differences are likely the result of the minor technical errors introduced into the Assay and Sample reagent master mixes and differences in automatic imager settings (i.e. image exposure time) on the Fluidigm platform. Though minor, these differences necessitate a method of normalization to ensure consistent genotyping across SNPs and IFC chips. Following these normalization procedures allowed us to increase accuracy by 21%. While not included in this study, future adaptations should also include RARatio-controls on each Fluidigm chip, which would include artificial DNA-pools that replicate the smallest RARatio possible. These controls are likely more meaningful than the full-BTS-controls that we used, particularly for model parameterization and training. If several of these synthesized RARatio-controls are included on each chip, then they can be included in the RF model training, enabling the use of hierarchical modeling, where IFC chip can be included as a random effect accounting for chip-specific differences within the model. It is unclear if this would remove the need for chip normalization, but we believe this would improve the accuracy of the method.

The pooled detection method outlined here has an overall genotyping accuracy of 98.5% for the 31 SNPs that passed quality filtering. This was verified using DNA-pools of wild CTS hybrids; comparing the Fluidigm and sequence data of the same samples yielded an overall accuracy of 96%. While there is a small degree of error inherent in the detection of non-native alleles at each locus, the chance of missing hybrids entirely is minimal in real-world scenarios. First, the likelihood of encountering a hybrid individual that is heterozygous for only 1/31 SNPS (i.e. 1/31 of its genome, or 3.2%) in any relatively recent hybrid invasion, including our study system in Sonoma CTS, is low. For this to occur, a full non-native adult (similar to the one detected in Sonoma in 2008) would need to backcross with full native CTS for at least 5 generations to contain non-native alleles in roughly 3% of its genome (each generation decreases the non-native fraction by 50%, (0.5)^5^ = 0.03). For Sonoma CTS with a generation time of approximately 4 years this would take a minimum of 24 years after the initial larval introduction. While such low non-native proportions may occur in other situations where non-native hybrids have been present for decades, it is unlikely in Sonoma County, where non-native alleles were first detected 15 years ago. In addition, past studies in the CTS/BTS hybrid system indicate that it is more likely that non-native hybrids have high fitness in native populations^27,28,30,31^, suggesting that each subsequent generation of hybrid offspring will have a greater chance of mating with other hybrid individuals, increasing the number of generations required to produce hybrids with a very low fraction of non-native genome.

Second, the probability of collecting and sequencing just a single hybrid offspring in a large sample should be low in wild populations, although it depends on the breeding structure of any particular population. For most endangered or declining species, we can often assume that the number of breeders in a local sample is small, and that sampling a reasonable number of offspring should ensure that multiple sibling group members are included in any sample, each of which should have, on average, the same proportion of non-native ancestry. This is certainly true in our Sonoma CTS test case. Past studies have demonstrated that the number of breeders in a given pond for a given year are quite low in small ponds. The CTS-wide average effective population size, or N_e_, is 30, with approximately only 1/3 of these emerging to breed in a given year^48,49^, and these numbers are presumably lower for the highly endangered Sonoma DPS. Even if only a single breeding adult contains non-native alleles, its offspring are likely to constitute a significant proportion of the larvae sampled from a pond. Our goal of detecting a single hybrid larva out of 75 individuals sampled per pond for a single non-native allele is an extreme ‘worst case scenario”, yet still returns a detection probability of 96–99%. If we increase either the frequency of hybrid larvae or the fraction of the genome with non-native alleles, there will be a much greater probability of detection.

In this study, we developed multiple validation methods to demonstrate the reliability of this novel approach, which are likely not necessary for future screening. Moving forward we recommend running pooled samples on the Fluidigm platform, re-assaying any candidate hybrid DNA-pools on a separate Fluidigm chip run, preferably with at least one DNA-pool technical replicate. If no BTS alleles are detected in these, we would conclude that the initial BTS detection in these suspect DNA-pools was erroneous. If the same candidate BTS alleles are detected in any of the re-assayed pools, we consider these to be true BTS detections and the individual tissues that comprise the DNA-pool should be extracted individually and either sequenced or run individually on the Fluidigm machine following the company’s standard protocol.

Finally, our study provides new insights into the potential invasion of non-native BTS into the critically endangered Sonoma DPS of the California tiger salamander. Given the biology of the salamander and introduction history coupled with the extensive verification and validation of these genotype data, we are confident that no BTS alleles were detected in this 2019–2020 Sonoma DPS sample. Given these results, there are two possible scenarios that describe the status of non-native alleles in the Sonoma DPS.

In scenario one, there are no non-native alleles currently present in the Sonoma DPS. This would, of course, be the optimistic conclusion for the long-term persistence and genomic integrity of Sonoma CTS. It is possible that the hybrid larvae detected in 2008 and 2014 were present in very low frequencies and eventually died out due to demographic stochasticity, or an inherent lack of fitness. This conclusion does not fit well with the results of previous CTS hybrid studies which suggest that hybrids have greater survival and fitness than native CTS^25,26,28,50^, although experimental lab crosses do indicate that first and second-generation crosses, and even later ones, demonstrate a wide range of fitnesses^51^. However, all of the previous studies focused on the hybrid swarm in Monterey County, which was the site of extensive BTS introductions between 1950 and 1960^52^. These case studies may not accurately represent the invasion dynamics of a distinctly different DPS with novel environmental conditions and introduction history. The cooler climate in Sonoma County may present a less hospitable environment for BTS hybrids, especially if hybrids are better adapted to heat tolerance^25,50^. Recent studies have also identified the influence that pond hydroperiod has on salamander fitness, where long duration ponds support larger, more robust populations of non-native salamanders^28^. The breeding ponds in Alton, where the two non-native genotypes were documented, have short, natural hydroperiods characteristic of seasonal vernal pools^53^, that may have provided sub-optimal conditions for hybrid establishment.

In scenario two, non-native alleles remain present, but at extremely low frequencies. Given the temporally restricted sampling that informed this current study, it is impossible to conclude that there are no non-native alleles on the landscape. CTS and CTS-BTS hybrids often remain underground for multiple years without emerging to breed. Therefore, hybrids may be present in the environment that did not emerge to breed in 2019 and 2020, and were therefore not detected in the exhaustive 2019 larval survey. This seems unlikely, given the overarching evidence from Central California on overall hybrid superiority, though continued surveillance will confirm this possibility.

## Conclusion

We have demonstrated the sensitivity of a novel, high-throughput genotyping assay, and used it to confirm that hybrids are not present in an endangered population segment. Although we did not detect any hybrid animals, additional surveys are likely necessary to ensure that hybrids are not present in low frequencies. Regardless, these results suggest that active management for non-native alleles is possible and has a high probability of success. We therefore recommend at least two additional years of sampling, similar to what was accomplished in this paper. If no hybrids are detected, we recommend regular surveys as part of an EDRR program so that novel invasions are rapidly identified, contained and removed. If hybrids are detected, we recommend enacting a targeted removal protocol, enabled by our rapid detection assay. Such a protocol may include capturing adult salamanders at breeding ponds, holding and genotyping all adults, and removing any individuals carrying non-native genes.

Although this method was specifically designed for Sonoma CTS, it could easily be expanded to include other CTS populations, and, with some initial optimization, to virtually any other taxon or system. In perhaps the most studied hybrid system, introduced rainbow trout presents a major threat to cutthroat persistence^16^. Previous studies have employed similar nanofluidic techniques^17^, but could benefit from the novel pooled-sample design presented in this study to rapidly screen larger populations to detect incipient invasions of primarily native streams. In other, non-model systems, our approach could drastically increase the capacity to identify hybrid populations and enable EDRR protocols. In the imperiled and highly endemic cyprinid fishes in Europe, multiple incidences of hybridization have been identified using conventional molecular tools^15^, though rapid surveys are not yet possible. Adoption of our method would require some initial sequencing of native and non-native genotypes to establish multiple genetic markers that are diagnostic between the species, but then these markers could be used to rapidly scan thousands of samples to map the extent of hybridization and to document recent invasions. We believe that this general approach opens the door to a wide variety of novel applications in conservation genomics.

## Supplementary Information

Supplementary Information.

## Data Availability

A full description of the genotype assay is included in the supplemental materials. Additional datasets generated and analyzed during the current study are available from the corresponding author on reasonable request.
