# Supplementary material for: A novel genetic strategy to enable rapid detection of rare non-native alleles
Source: Sci Rep. 2024 Oct 29;14:26027. doi: 10.1038/s41598-024-76149-8 (PMC11522522; doi:10.1038/s41598-024-76149-8)
Supplement: Supplementary file 1 — Supplementary Information. [file 41598_2024_76149_MOESM1_ESM.docx]

**Methods**

*Experimental Optimization*

We performed several experimental Fluidigm assays to optimize our detection of BTS alleles while minimizing the number of assays to reduce cost and time. We determined the optimum amount of total input DNA by running Fluidigm assays on a range of input DNA. The Fluidigm protocol suggests using 12.5ng of DNA for human genome samples, which amounts to 3,619 genome copies assuming a 3.2Gb genome. The axolotl, which is a close relative of CTS also in the genus *Ambystoma*, has a 32Gb genome^1,2^, approximately 10x larger than the human genome. This would require 125ng of total DNA to achieve the same 3,619 genome copies. We therefore assayed input levels spanning this range, using 0ng, 12.5ng, 25ng, 50ng, and 125ng of total input DNA. We identified the optimal amount of input DNA as the treatment that elicited the greatest positive effect on X and Y allele intensity in known heterozygous pools. We performed a linear mixed model with the RARatio (See Description Below) as the random effect and selected the total DNA value with the greatest effect on allele intensity.

To reduce the cost and labor required to scan thousands of samples, we pooled larval tissue samples prior to extraction. We performed experimental assays to identify the greatest number of individuals that could be pooled, while still providing sufficient sensitivity to detect one BTS allele in the pool. We define this value, the number of BTS allelic copies in a pool of CTS alleles, as the Rare Allele Ratio (“RARatio”). For example at a given locus, one heterozygous larva (1 BTS and 1 CTS allele) combined with 14 homozygous CTS larvae (2 CTS alleles each, 28 CTS alleles total) would amount to a RARatio of 1 in 30, or $0.0\bar{3}$. We created these experimental pools by pipetting an exact amount of homozygous BTS DNA extract with an exact amount of homozygous CTS DNA extract to achieve the desired RARatio. In the example above, we would combine 1 ng of BTS DNA with 29 ng of CTS DNA to achieve an RARatio of 1/30 or $0.0\bar{3}$.

We tested a range of RARatios including BTS:CTS allelic ratios of: 0, 1:80, 1:40, 1:30, 1:20, 1:10, 1:5. We identified the optimal RARatio as the lowest RARatio where we were consistently able to identify the presence of the rare BTS allele. Our first analysis examined the accuracy of genotype calls using the Fluidigm Genotyping software. While useful for relative performance, we note that the software is not designed to detect rare alleles in pooled samples, since it expects an individual to have, at minimum, a 1:1 ratio of alleles if the individual is heterozygous at a locus. We therefore developed a custom method for detecting rare alleles using a random forest (RF) model from the software package randomForest (v4.6-14)^3^. This method is similar to the one described in the “Random Forest Custom Genotype Calls” section in the main text. We constructed an assay-specific random forest model for each RARatio (rounded to the nearest 0.01), and trained the RF using 80% of the available data, retaining 20% of the data for model evaluation. We calculated the average Out of Box (OOB) error for each SNP and RARatio combination by taking the mean of the OOB error for each random forest iteration. We also estimated the sensitivity of each SNP-RARatio model by dividing the number of pools where BTS alleles were identified by the total number of pools that contained BTS alleles (Observed / Expected). We selected the lowest RARatio that had an OOB error less than 10% and a sensitivity greater than 95% (Figure SI.1).

**Results**

*Experimental Optimization*

Results from the experiment comparing the mean allele intensities across a range of input DNA suggest an optimum of 50 ng of DNA. The coefficients for the effect of total input DNA on Allele X intensity were 12.5ng = 0.6982, 25ng = 0.7059, **50ng = 0.7151**, and 125ng = 0.6429. For Allele Y the coefficients were 12.5ng = 0.5116, 25ng = 0.5695, **50ng = 0.5983**, and 125ng = 0.5099. However, the effect on X and Y allele intensity appeared to be fairly similar with input DNA between 12.5 and 50 ng. Assays using the most input DNA (125 ng) yielded the lowest allelic intensity, and therefore should be avoided.

Across the range of RARatios examined, we identified a pool size of 15 individuals (RARatio = 1/30 or $0.0\bar{3}$) as providing the optimal trade-off between accuracy and scalability. Using the results from the Fluidigm Software, we visually identified diminishing returns for RARatios greater than 0.03 (Figure SI.1). The mean probability of correct software calls for RARatio 0.025, **0.030**, 0.036, 0.050 are 0.68, **0.87**, 0.86, 0.72, respectively. The RF model custom genotype calls corroborate this pattern; an RARatio of 0.03 represents the largest pool size (or lowest RARatio) with a median OOB error of less than 10% (0.025 = 17.6%, **0.030 = 8.8%**, 0.05 = 4.8%; See SI, Figure SI.2A), and also a mean sensitivity greater than 90% (0.025 = 85.5%, **0.030 = 94.7%**, 0.05 = 94.8%; See SI, Figure SI.2B).

**Figures**

| **SNP_NAME** | **ALLELE** | **ASP1_NAME** | **ASP2_NAME** | **SNP_SEQ** |
| --- | --- | --- | --- | --- |
| 015_chr10x_s2_101553022 | GA | G | A | AGAAGGAAGAAGAAAAGAAGCCACACGTCAAGAAGCCTCTGAACGCATTCATGTTGTACATGAAGGAAATGAGGGCAAA[G/A]GTTGTGGCAGAGTGCACGCTCAAGGAAAGTGCTGCCATTAACCAGATCCTCGGTAGAAGGGTAAGATCTCCCTTCCTTTCTCCGGTGCTCCTTTGACTTAATCACTGTTT |
| 022_chr10x_s2_272909334 | TC | T | C | CATTCCGTACAAAGTTACCTTTCAATGATCACCTTTATTGCAAGAGGCCACAAGAGGACCCGTCATCAGAAGATGGGTCGGT[T/C]GCATCAGAGAAGCAAGATGGAGACGTTCAATCGGAAAAGCAAGATGGAATCAAAAATGGTCAAAAAATCTCTCTTGTTCCAGATCAACAGTTAACTG |
| 041_chr10x_s3_333275042 | CT | C | T | TTTCTTTCTCTTTAAGATGCGGAAACCAAGACCACAGCGGGAGAAGGCTCAGTGGGAGATTGGAATTGCCCATGCTGAAAAAGCACTTAGAATGAGCAGAGAAGATAGAATCGAGCAATA[C/T]ACCTTCATTTATGTGGACGAGGAGCCTGAGGATTTTCCTGAAACAAAAAAGACTGCTGCCACTAAAGTGGAGACTAAAAAGACTCGAAGATCAAAGTCTGTCTCAAGCAGTGAGCCAGA |
| 064_chr11x_s1_338550742 | AC | A | C | ATTCTGATCTTTATTAAAGCTTCCCAACTTTTTGGTCGCCTAGTGACACACCGTTGTATAACCTGGAGCCCTGTGAGCCGATGCCCTTTGACGTCTCCAGATTCCGTGGGCTAACTGCTGC[A/C]GTGCTACTGGATCTTACCTTTCTTACTGGCATCCATGAGGATTTGGGAAAGCAGAATGCCAAGCGACATGATAAGAAACACAGACATGAATCTGAAGATAAATGTGATGCAGAGCAGAA |
| 076_chr11x_s2_101678172 | TG | T | G | ACACAACTGTCCTTGTAAACAGTGCCCTTTCTTCCATGATCCACAGGAAGCAGAGCAATGTCACGGCAGCGTATTCGTCACTGCCCCCTGAAGCACTCTTTGCAAATAGCCTTGGGATGCT[T/G]GTTGGAGTTTTTGGTTTGCTGGTGCTCTGGATGCTATTACAGACGTCATGGCAGCGCCCAAATCAGTGGGTATCTGAAGCACGGCAGGTAAATCAACACACATTAACAGCTATGGGTGG |
| 092_chr11x_s2_430644556 | GA | G | A | GCATGCCAGATGTCCAGAACTTTAGGGAAACTATCCAGCAATTAGAAGGCCGCTTAGTGAGGCAGGACCACCAGATCCGAGAGTTGATTGCCAAAATGGAAACCCAGAGCAATCAGGTGAC[G/A]GATATCAAGCGCACTATACGTAGCTTGGAGGACAAAGTAATTGAGCTGGAGGCACAGCAATGCAATGGCATCTTCATCTGGAAAATTGAGAATTTTAGTATGCATCTGAAAACTCAAGA |
| 127_chr12x_s1_390748989 | GA | G | A | TGTCAGGCATTACATCTCAGTCCTTGACTTCTCCTCCTTCACAAAATATTCCAACCTACACCACACA[G/A]GCCTCTATCTTCCCACCTCTTGTCCCTAGTATTCATTCACATGCTCCTGTGCAGCCCATTGTCAGTACGCCTGCCCCATGTTTCACAAGCACAGTAGTTGTTGGGTCTTTATCAGTCCC |
| 129_chr12x_s1_447766070 | GC | G | C | AGGGTGAGATTGGGGAAAGGGAGGGCGAGTAGGTGGCAACTCCTGCCAGTCTGTCCATTCGTGCTTCAATGACGATCCAGTGCTGAACTTTGTAGAAGATCTGTGGGGGCTTTACTGGGGG[G/C]TCTGAAGGCTGTCTGGAAACCTGGGGGAGGAGAATGAAAGCTGGTGGGGTTTGTGTGGGGTGGGTAAGGATTCCAGCCAGCTCTGTGCAGGGGGATCTGTGCGCTGAAGGGGTTCATGT |
| 155_chr12x_s3_067505482 | TC | T | C | ATGACCCAACCACTCATTTTGAGACTACATGTGATGACATCAAAGACATCTACAAGAGGATGACTGGATCACAATTTGACTTTGATCAAATGAAGCGCAAAAAGAGTGACATCTTTGGAGA[T/C]GAACAGCAATAAACTACTGTGCATGTAGGAGGGATGAAAGTTGGCTAATACAAAGATGTTTCAGGAACTTAATGAACACTGTATAAAATTGGGTATTGTAAGCCTGCTTTTGTAAACTC |
| 159_chr13x_s1_293454629 | TC | T | C | TTGGTGATACTAGGCTGCTTAGTGGAGGACTGAGACTGGCTGGCGACGGTTGTCACAGTGGTGTACTGTGGAAGGCTGGTAGTCACAACAACTGGTGGTGAAAGTCTAGGCATCTCAGGCA[T/C]TGGGTTGGAAGGCTGTGGGGGATCCAACTCTGCTTCATCCACTGTGGTCTGAAGAAAGAAAGAAATAAGTATCAAGATAAAGAAAAACAAATAAACCAAGTCTGTG |
| 166_chr13x_s1_392141078 | TC | T | C | GCATGGCATCAGGAGAGATCACTGCTGAGTCAATGTACGATAAAGAGGAATC[T/C]GTGTTTAGAGAGTACTTCGCATTGGAGGACTCCAAGTTCAGCCGGTTCAATTCCTGAAAATCAATAACCATCAATCAACTTTCAAGCAGT |
| 177_chr14x_s1_148700382 | GT | G | T | TACATTTGTAATGGGTTTGTGTAAATTAGATGTTTTGATGGCTGATATAACAAAGTGGTGTTAATACTTGACAAAGTTCTGCAAGTAGAATCAAATGATTTACCATTGTTTGAACTAATCT[G/T]GAGCAGTGTGTGGTGTTAGATTATTGCCTACAAAACTGGGTCTTAGAAAGGTATGACTGTATAGCTGATGCTATGGCACAAAA |
| 228_chr01P_s2_426088410 | AG | A | G | AGTTCTGCCGGAGTCTGAGCAATGTTCACGGCTTCCATCTGTTCAAAACTCCCCAATGGATGGCACTTTGGCACCAGTTC[A/G]GAAATGGAGCGCTGGTGTAGGGTGCCAGTGATCAGCAAAATCACATTGTCAATCATGTAGCTGTATCTGGCAAAGAAAGAGCAAGGTGGTAAGCAACAGACAAAACCACAAGCA |
| 243_chr01P_s3_419787534 | CT | C | T | GTGTAAGATCAGACTAAACCATGAAATAAGCACAGTAAACTTTACTTCATCCCTAATGGTTTTTAATTTTATGGATATTCCCAAATTTGAAATAATATTATCTTCTT[C/T]ATGGTAATGAACATGCCTATTCTGTGATGATTGTTGTTTATATGCAACGAAAAATTCCAGATTTTAAAAAGAGTTGACTTTTTGGTCCAGTTTGGTTCCTTAACTATTGCCAATACATA |
| 253_chr01Q_s1_275942258 | TC | T | C | TGTGGCTGCTCGGATTTTTCTATTTCGGCTCACTGCCCCTTGGGATCTATTTATTGGTGATTCAAAAAGTGACACTAGGAATCATTTG[T/C]GTCAGCCTGGTTCCGTCCAGCCTAACTGTGTGTCTTGTTTATGGATTTTGTCAGTGCCTCTGCCAGGGTATATGCGACTGCTCTGCGAGGAACTGATGAGTAGT |
| 263_chr01Q_s2_159414134 | CT | C | T | TATATATTGTTCCAAAGAAATAACTAGATAGCAAACCTGGTTTGAGTTTATCCTTGAGGTATGGGACCAGATTGTATTCCTTGAGCACAAGCTCCCAGTCTAAATCAGGAATTGTCAAATT[C/T]GCAAGAGTCCCAAGGCATTCTATCACAAATTCCTCCTCTTCTTCATTGGAAATTTGAGCTGCCAGATCACCAACATAATCCTGGAGAAAACATAATCACATTAACCTTTGACTGCAT |
| 266_chr01Q_s2_408777327 | AG | A | G | CATCATTTGTCAAAAAACAGTTACCTAGTTCCTTCATTCCACAAGGCTGACTGAGGTCTTTATTGCCATCTTCCATGTGTGCAGTTTGCACCAACCCTTTTAACGAGGCACAATTTAGAAA[A/G]CAATTTGTGCTGTCATTCAGAATACCCGACCACTTTTCTTCTTCGGTTTCCTCCCTTTGTGTGTTCTGTTTTTTGG |
| 303_chr02P_s2_069466403 | AT | A | T | GTCCTGTAGAGTCTCTGCGAGCGTGGGGCATTTCTTTCCATCGTAAATGACAAGAAGGGAGCTGGAGTAAAAGCGGTAGGAAACCTGTCTCTCCAGGACATCCAATAGACCACGGAGCTTG[A/T]AGAGGATTGGGTCAAACAGGTCCTTGCGCAGGTCCACACCATTGTGGAGATACTGGAAAAGAGCGTGTTGAAAACCATCGATGGAGAGCCCACGTCCATAGTATTTGTTGCGACATACG |
| 340_chr02Q_s1_373362924 | GA | G | A | AGAATGCACTACTTGCTGGTCCAATAGAGGTTACTGTATAGAGGGAGATTAAAGCAAGCATTCAGATTTGGATGGAGCGA[G/A]GTATGGGTTAAATTATATATAAATGTTATATTTGTTATGACATTTTTGACAGATTCAGTTCCAGGAACATTCAAAATTTAAATTTGTGTCCATTGTAACACAATGTTCACATAGTTGAA |
| 357_chr02Q_s2_246374865 | CT | C | T | CTGAAATTAGTATTACAAAAATATGGCAACACAAAATGGTTCAATAAAAGTGAACTACAGTAAGGCACGATTGTTCTGTATTCAGAAAAGTCAGGTGTCTTCTAACAAGGCAAAAGGGCCC[C/T]GTCAAGTGCTAAATATTGCATTTTAATTAAAATGTCTGAGTTGAGAATAAAACTTTTCATATAATTAAAATCTTTCAAGGATTT |
| 361_chr02Q_s2_399916527 | AG | A | G | AGTCGAAGGGGGACATAAGAGGGAGTCAATTCAGGGGAAAATACACGATGGATTTGTGAAGCCCTTGAACTGTAGATTTCTTGTAGATGTATCCTTCACGTTGTAAATATGTTTTGTAGAA[A/G]TGGAGCCATGGGAAGCCATGTGTATCGGAGCTTTGACATCCAAAACTAATCAATGCTAAGGTGGCTAAAAACCAAGCCTTTTACATGTAAACTTGTCTACAGAATTTGCTTTTGTTTAT |
| 421_chr03Q_s1_016523147 | AT | A | T | CCGTTGGGATGAATCGTGTCTTTTTCGGTGAAACTGTTGGTGAAAGACCCCAGTGCAGCAAACTTACTTGAAGCCATCTTCTGAAACCCCAATGTGAGTAGTGAGAAGTGAAAAAACAAGT[A/T]AACTTTATTGTTAAATATATGTTTATGGTCACATAAACTGCCCAGTAGCTTACGACACAAGGTTATTTACAGAAACAACGGCAGGGACATTGGAAAAAAGGCACTTTCAGTAGTACAGT |
| 425_chr03Q_s1_139827286 | TC | T | C | CCTGAAAAGGAAAAACAAGAGCGAATAGAGGAAGGAAGAAGAGAGCGAGCAAAAAAACTAAAAGATGTCAGGGAATCTAAGTGTTGAACAATTTACATA[T/C]TCAAATTCACAAGCAAAGATTGTTTACAGACATCATGAATATATCAATGCAGAACAGCTCTGGAGATACTTACCTGAAGATATATACCAAACGTAGTCTGCTTCCCATGTGACATTGTT |
| 438_chr03Q_s2_012999827 | GA | G | A | GGAGCATCCGGCAACTTACTGAGTCCTAAAAAAGATGAAGTCACTGAAATGCCTTTAAAAGAAAGAAAGCGAGTGAGAATCATGGATGATCAAAATGAATCCC[G/A]AGCTTTGCATGAAAGTAACGGATGTGAAAACGCCAAAATACCTACTTCCCCAAGGTCAGTGTCTGCTTCAGCATCTGGTGAAGGAATTCAGCAAAGGCCTACATC |
| 455_chr04P_s1_393441828 | AG | A | G | CAAGAGTATTATTCCTACGTCTCCTACAAGATTAACCTCGAATTTCTCGACAACTGAAATTAACCAAGCCTCCATCTTTGCTTTAGCCGAAATCTGGGACCAT[A/G]CTTAAGAAAACTAACAGTGTAAATCACTCTGTATTTTAAGAGATTTTGACAAGAATAAATTACTGCAGAATGTACACTGTGTGTTTTACAAAATCCGCCATTTTTCTCTATCAAAACGT |
| 465_chr04P_s2_187207589 | AC | A | C | GGATTTTAATGTCAAAATCTTTGAACCAGAGGAAGTGCAAGGTTGTTCAGACACAGCTTTTTCTGGCAGATGCCTATTACTTCTTGT[A/C]CGGGTTAGTGAGGCAGACTCTGTTCCTTTGCCATTCACGTGAGCAGCAGTGATTTTCTGTGCTGCCGAACGAGCTAGTGTTGTAGGTGGCAAAGAGGTAGCATTCTCTGTCTTTGTCTG |
| 476_chr04P_s2_487436412 | CT | C | T | GTCTAACCTCCTGGGAAAGGCGCATGCAACATAATGACTCCACTGAGAAGGCAACCACATACACTCCTCAGGTCCCAAAAAAGCTCTCTTACTCCGAAAAGAACAAATG[C/T]GCCTCCTTGGAGGAAATCCTGTCTCGCTCGGACTCTGCACACAGGGCGGTACTGAGGAAGAGCGTAGAGACCCACCTCAGCTCCACAGAGTCCGAGCAGCTGGCACGGATACAGGAACT |
| 485_chr04Q_s1_063106804 | GT | G | T | ACTGGAAAACATTTCTGTGTGGGTTCCACCTGTTGCTCCATGTCTCTATATTTTTTCTAAGCTATTAATTAGGC[G/T]TCTCAAAGTACAATGTTTATATACCAGATTCCTCTCAAACAACAGACATGGTACTTTCTGGATTCACCCTAATCATCCTAGAAGCATTACGGAGTTCATGCAATTGTTTGGCAGGCTTG |
| 492_chr04Q_s1_247455206 | TG | T | G | TCTCCTTGGGGATAGACCTGGTGCACGAGATAATCAAGGAAGGTCAGTACCAGAGTTGCACGAAGCCCATGGGCAACAAGAAACCGATGCAAAAGAAGTTGATGAGTGGCCTGAAGAAGAT[T/G]CACAAAAAGGTCTCGACTGGCCTCAATCTGATGCACAAGAACAACAGGATTGGTTAGGTTCTGAAGCAAAGGAAGGCCCCAGTTGGGCAGGATCAGATGC |
| 536_chr05P_s1_142916477 | TC | T | C | ACTGTATAGTTTGGTTTGTCTCCTTTTAGCTGCCAAACTGTAGTCTGTAGTAGCTTTGTTTGTTTTTGGTTTAATACCATTTTAGCTGTTTCAATGATGTTTCTTGTAATCTTCTTACACA[T/C]TTGAGTGCAGGAGAGTTCAAAATGTTTTCAATGAAAACAAGCCCTTCCAATTACAACTCCAGACATCCTGGTGAATGCTCATACAGTATGCCTTACTTTTAGTAATCATGAAACCATCA |
| 537_chr05P_s1_244223291 | AT | A | T | GTGGACATGTCGGATACAATGTACCCTAGAAACCCTGCCATGTGCAATGAGGCAGCAAGACTAGCA[A/T]CATTCATTAGTTGGCCGGATTACTCCCCAGTAAAGCCAAAGGAACTTGCACATGCTGGACTTTATTACAAAGGCATTGCTGACCAAGTGGAGTGCTTTTGTTGTGGTGGC |
| 538_chr05P_s1_256651842 | CT | C | T | GGTTACTTGCAAAATAGTTACTTGATGCGTTTCCAGTTTTCTTTTGTCCTCCATCTACTGTTTTGGGGCAGTATTTTGTATTTCAGTAATTTACATTATAAGCTTTTAATTTTAATTGTGT[C/T]ATACACAGTGGTGCTTATAAATGTAGCTTTACAAAGGGTTGTGTAGAAATATGTACACTTTTACATGCC |
| 563_chr05Q_s1_145394827 | CT | C | T | GAGGAACTCCTGCTAGGAGGAGAGGGAAGGCTGCTATCGCCCAACAAGCTTGAAGCAAGTGGAGAAAAGGCAAGACCAGATCCTTTAGCCAACTGCAATGGAAAAGGATGTGTCTCCATGA[C/T]GGGCTGCCCAGTACATCTTTCAGCTCCAATTGCCATGGTATGGGAAGGGTCATAGCACATGTGAACGCCAGAGCTTGGAGTATGTGGTGGAGAGTGCTTAAAGTGGAATAAAGGAAATC |
| 590_chr05Q_s3_008288909 | AC | A | C | CTGTTTAAGACATGAATGATTTTATTTTAATTATGTAACTCAATGCAGCTTTAGAGTTCCTTTTATGAAGACTTGGTAGGATGCTGTTTGTTCTTCCATTCATCTATTATTTGTAATC[A/C]ACTTTATTTTTGAAAGTAGGTCGATATACTACAGACTTGGTGAACATTAAACTGTTCATATTCTCAACTTGTACATTTTATTTACTTTCATATGTTATTAA |
| 595_chr06P_s1_149521203 | GA | G | A | AATGATCACAATGGCCTTCCTTAGCAATACCAAGACAAATGGAACCCCAGCACAGGTGCTGCACACTTAAGGACTGTACTGAAGGATGAGCATACAAATCCCATATGGCATCGGCTAGAAC[G/A]TCATCATACTTGTTCTTCATTCTGCATGGCAGGCAGCACTGTTCTGATATTGTTAACTGCTTTATCCCATGGCACTGGACCTACAAAAGGTATCATCACAGACGACCATGCACTTGGCA |
| 616_chr06P_s2_206254159 | TC | T | C | GTGTGTAGGCAGTGTTTGATCCAGCATTTCGTGATTCAGTCCAGCGTACTTTGACGTGCCCCCGTCCATGGATCTTCAGAGACTTCACCCTTATTTCCCCGGTAACTTCCAGATTCACCCT[T/C]CCTGAGACCGTGTCCCCGCTAGAGTACACGGGGACGTTGCTGTCATTGAGACAGTCGAAGGCTATTGTCAAACTCTTCACCTTCCCCAGCACCATTTTTGTAAC |
| 630_chr06P_s3_274235370 | GA | G | A | GTGGATGACCATAAACATCTCTGCTCCTCGACCTGCCATGATCACGGCTTCTTGACCTTGACCCATCTCTTGATAGGGAGCTCCCACTAGTTATAGACACTGTCTGGCTTCTCATTGATGA[G/A]CTTCGATCTCTGCTTCTTACTCGTTTTTTCCCACCATCTTTTTGTTTTATCCTCTCCCGGCTTCTTGAACTTCTGCTTTTCAACCTCTCTTTGCTGTGGCGCTTTTTCCT |
| 638_chr06P_s3_459994470 | GA | G | A | GGGATAGTTCAATACAGCATTGCTTGGGCTGGTTAACATGTAACAATTGAATCAATACAAATCAATGAATCTTCAAAAT[G/A]TGTATTAACCATTAAATAAAGCCACAAAAGTCATTAATATGAGGTTAGGGAGAAAGCAGATATGGCAGGGCACGCAGTGACAATAAAAGAGTAGTAGGTAGTGAAAAGAGGTGTTCGCA |
| 657_chr06Q_s1_332970966 | AG | A | G | CCCACTTCGGGAATAACATTGTGCGATGATACAAAATAGCTGTCAGAGTAATCTTCCCTGTAAAGGTTATTGACATGAAGTGGAGTGGTTTTATTCTGCACTTGAGAGTAATTTCCTGGTG[A/G]AGCACTGTAGTTTGGAGGGTAAACTCCATACCCAGATGACGGCAGCCGATTCAAAACTGGACCTGAAGAAAATAAGAAAACCAGAAAATCAGACAGTTAAGAGGCTTCTTAATTCAACA |
| 670_chr06Q_s3_115002488 | CA | C | A | ACTGTTACTTTTAAATGTATTTCTTTGATGTTTACTTTTTTTTCTTCTCAAAAGGTAAAAAGAAAACCTGCTAGAAAACAGAGGAAGTAAATGGCCAAATGTAAAGGCATGACTTGAGCTT[C/A]TGCACAAACTGGGATTCACTTGTTTCCTGTAGAAAAACTGAACTTTCTTCAAAACTACCGCTTTCTGGCCTGAAGACAATGCTTCCTTTTTAAATAAATTTTACTCCACAGAAATGTCG |
| 715_chr07x_s2_107318852 | CT | C | T | GCCACGGACAGACCAACATTTTGGTGTACATAACAATCTGTTCAGTTATTGGAGCTTTGTCCGTTTCTTGCGTGAAAGGCCTGGGCATTGCTATAAAAGGACTCCTTGCAGGCAAGCCTGT[C/T]CTTAGCCACCCCTTGTCCTGGATCCTGGTGCTAAGCCTTGTGATTTGTGTCAGCACACAGATCAATTATTTGAACCGAGCTCTGGACATATTCAACACCTCCTTAGTGACTCCAATATA |
| 728_chr07x_s2_362360202 | CT | C | T | AGTTGCACTCTAGAATACACAGGAATAATGATTTTCCAACAAATAAATTGAGCTTACCATATGAAATCGGCCAACACCACTCCTGAAAGGCTGCCTCATGAACCGGCTTGGTTTACGAAAC[C/T]CTTCTGTTTTAGGACTCCGAAATTCTGGGAAACTAGATCTTTCTTTCTCCCTGATTTGATACACAA |
| 741_chr07x_s4_028510156 | CT | C | T | ACTGTGCTTTATTGTAAATCACTTTTTGGCAAAGTTCAGCCTGAAGAGTGGTGAACATTGAAGGTTGGGCTAAGAAAATAGAACCTGAGGGAAACTGACAATCAGTCAATGACAAGCAACA[C/T]GTTCTAAAACGTTCATTTAGTTTGTTAAATATTTCTTTTCATAGTTATACTTCTTACATTATGAAAAAAGGATTTATAGCTCTTGGCATTCTACTTTCACTGTTTTTCTTCTGCATTTA |
| 747_chr07x_s4_130875402 | CT | C | T | GGTTGACATCAGACATCTCAAAGAACTCTGAGTCTGGTTACATCCTCTTCTACCAGTCTCGGGACTGAATGGGAAACTCGATTGAGAGACACTTTCTGCCTCATTTCTTCTCTGGTTATTT[C/T]CGGAAAAGGAGCAAGCACTGATTAAAAGACAGAAAAGAGGAATGCAGTGAGCTCAGGGGGCAGTAGCGCAGTTTGCACACTCAAAGCAAAGAGCATGGATCCAGCGGAAGAAGTATGTT |
| 777_chr08x_s2_020500015 | TC | T | C | CAATGCACTCACATTTTGTCCACCAGCCAACTCCCAGGTGTAGTTCCCGTACTGGTAGACCATGCAGCGAGACTGGTCCTTGAAAAG[T/C]GTAGCGGAAAAGTTGCTCCACCAGTCGAACATATTACCATCTTTATCAAAATTTCTACCTGTCCGTTAAAAGAGAAGCGTAGAGAGTTGGAGCATTTTGGTAGACAGGCA |
| 789_chr08x_s2_220216922 | TG | T | G | TTTTGATATATGATGTGACTTTAATCGTCAGGGGTATTTTATCTGCTTTTAAATATCAAAACCTGAAATGAACTTAGATGAAAAGCTTAACATAAAGTTAAAGATGGTTTCTGTTTTT[T/G]AGCTAAAATGCTTTCATGTACACTGTCAGTATTTTAAACCAGAAAGAGATCTGAAACGTGTTTTGTGAGTTTGTATTACTCCACAGAATCACTGTCACAACA |
| 806_chr08x_s3_268401213 | TA | T | A | CACTGCATTAGGATTTCCCTTATTTCTGGATTCAGGTGTTCAACAGCCTTTGCAGCTTCAGTAATGGCTTTCCGATACAATCCTTTCTTCTTTCGATTAAATCTCAAATTGAAAAATTCAC[T/A]GAAGGAAGAAAGTTTTGAGACTGATAGAAAAGATGTTGTTGGGTTACCGAACCATGACACTTTTGCTTCTTGCCAGCAGGGCTCTCCATTTTCCTTGTGACTGGTACTAATACTAAGAA |
| 824_chr09x_s1_324570600 | TC | T | C | CCCAGAAGGACTACTCCACTCTCCACAGAAATGAGGGAAAGAACACCTTCTCACACACTTAAGGACATTGAAGCACGATAAATGTTGATGCTGCGTTGGAACATTTTACAATGAC[T/C]GTATAAGCAAAAACATATGTAAAGTCATGGACAGACTGGGACATGTTAATTGTAAAAAATGTACATGCCTCCAGGTAAACATTTTGAACTTTTTTGTACATTGTATGTCGAAAACGTAC |

Table SI.1: Table of all SNPs designed for the diagnostic CTS Panel. All reference alleles (ASP1) are CTS and alternate alleles are BTS (ASP2).

Figure SI.2: Accuracy of the Fluidigm Genotyping software across a range of RARatios. Panel A shows the probability that the software makes an accurate genotype call. Points represent an accurate or inaccurate detection of a BTS allele at each RARatio. The blue line depicts the logistic model that best describes the probability of a correct call. Panel B shows the proportion of software genotype calls that were correct or incorrect for each RARatio. Note that because this software is not designed to detect rare alleles in a pool of individuals, these figures should be used to compare the relative accuracy of various pool sizes but the absolute metrics should not be used for further interpretation.

Figure SI.3: Accuracy of the Random Forest (RF) custom genotype calls across a range of RARatios. Top panel shows the RF Out of Bag Error which is an estimate of the error in each SNP-specific model. Bottom panel shows the sensitivity of the model to detect a rare BTS allele in a pool of CTS. Sensitivity is estimated using a subset of the data that is excluded when training the RF model.

Table SI.4: Table of potential BTS alleles detected in all assays. Assays include: 1) The original fluidigm DNA-pools including all field samples of unknown genotype which were analyzed using the custom random forest model. 2) A rerun of all DNA-pools following the same protocol as above. 3) All suspect DNA-pools were re-extracted as individual tissues and run on the fluidigm imager using the original software. 4) STA amplicon sequencing which provided high-depth sequence reads for each assay, enabling counts of BTS vs CTS alleles at all SNP loci.

Literature Cited:

1. Nowoshilow, S. *et al.* The axolotl genome and the evolution of key tissue formation regulators. *Nature* **554**, 50–55 (2018).

2. Smith, J. J. *et al.* A chromosome-scale assembly of the axolotl genome. *Genome Res.* **29**, 317–324 (2019).

3. Liaw, A. & Wiener, M. Classification and regression by randomForest. *R News* **2**, 18–22 (2002).
